# Healthy Volunteers for Clinical Trials in Resource-Poor Settings: National Registries Can Address Ethical and Safety Concerns

**DOI:** 10.1017/S0963180118000476

**Published:** 2019-01

**Authors:** FRANCOIS BOMPART

## Abstract

Healthy volunteers (HVs) who participate in clinical trials are a vulnerable group that deserves specific protection. We assessed the number and types of studies that involve HVs around the world and outline the methodological barriers to their analysis. We found that tens of thousands of HVs are involved every year in clinical trials in a large variety of countries and that the overwhelming majority of studies are not “first-in-human” but pharmacokinetic studies. The two cornerstones for both ethical and safe participation of HVs in clinical trials are properly obtained informed consent and a minimization of exposure to risk, in particular by avoiding concealed participation in multiple trials. To minimize the risk of exploitation of HVs and their exposure to risk, we propose ways to ensure genuine informed consent, and advocate setting up national healthy volunteer registries as established in France and the U.K.

## Introduction

The World Medical Association’s Declaration of Helsinki^2^ states that “some groups and individuals are particularly vulnerable and may have an increased likelihood of being wronged or of incurring additional harm. All vulnerable groups and individuals should receive specifically considered protection.” Healthy volunteers (HVs) who participate in clinical trials constitute a potentially vulnerable set of persons that deserve the “specifically considered protection” recommended by the Declaration. Unlike patients, who may expect some direct health benefits from their participation in a clinical trial, HVs cannot expect benefits other than financial ones. In health-related research, “resource-limited settings” are not limited to low-income countries but can exist within middle- and high-income countries, as acknowledged by the 2016 Council for International Organizations of Medical Sciences *International Ethical Guidelines for Health-Related Research Involving Humans*.^3^ In these settings, HVs are most often poor people with low literacy levels who may not be able to understand the risks they may be taking. In addition, they often are in no position to refuse financial incentives, and indeed may be actively seeking them, because for many of them, participation in clinical trials is a critical source of income. This paper aims to summarize available data on studies in HVs, outline the key ethical issues that relate to HVs’ participation in clinical trials, particularly in resource-poor settings, and propose ways to address these issues.

## Healthy Volunteers in Resource-Poor Settings

A variety of reasons may motivate participation of HVs in clinical trials, such as altruism, accessing ancillary healthcare benefits (e.g., a medical checkup), scientific interest, curiosity, meeting people, etc. However, financial gain is the primary reason why HVs participate in clinical trials. This has been well documented both in the U.S.^4^ and in Europe^5^ for “repeat volunteers,” i.e., those who have participated in several clinical trials, and also by researchers in India,^6^ Brazil,^7^ Romania,^8^ and Portugal.^9^ Motivations can vary based on demographic or socioeconomic characteristics, for instance, financial gains can be the main reason for participating for younger people,^10^ but may be seen as of secondary importance by medical students.^11^

Resource-poor settings, i.e., difficult economic conditions, low literacy levels, limited access to healthcare, etc., can be found in low- and middle-income countries, but also in high-income countries, as was well documented by U.S. teams.^12^ Some U.S. authors have described how economically disenfranchised people are systematically used in pharmaceutical development.^13^ Others, describing the population of healthy people who volunteer for participation in clinical trials, have spoken of the “exploitation of a research underclass.”^14^ Outside of the United States, little research seems to have been carried out to document the characteristics of HV populations who participate in clinical trials. There is little doubt that in many settings, HVs can be characterized as a specifically vulnerable population that is exposed to a risk of exploitation, because they may not be able to provide genuine informed consent for participating in a clinical trial.

The ability to provide informed consent rests on two pillars: the ability to properly understand the potential risks and benefits related to the proposed trial, and the ability to refuse participation. On both accounts, HVs in resource-poor settings are vulnerable: their literacy levels may not enable them to properly understand what risks they may expose themselves to, and the financial compensation provided for their participation can become an undue financial inducement if they are in no position to refuse it. Rabin et al.^15^ have described how 58% of a panel of HVs in Israel did not even try to form a proper understanding of potential risks and benefits on which to base a “quality decision” to participate in a trial. Similar observations were made in India,^16^ where a study found that most HVs had decided to participate before being offered any information during the informed consent process, or even before visiting the study centers. One of the few available papers on HVs in low- and middle-income countries shows how, in India, contract research organizations (CROs) resort to “middlemen” to recruit poor participants who have no understanding of what the studies are about and basically “chose to participate in the trials due to insufficient income and unstable jobs.”^17^

Several U.S. researchers have investigated “professional healthy volunteers,” who make a living out of their participation in multiple clinical trials. Payments of several thousand dollars for participation in a clinical trial are not unusual, which can lead in some cases to yearly incomes of over $30,000.^18^ Several authors have described the tactics that some have developed to optimize their chances of being selected for the most appealing studies, most often those that offer the best financial benefits^.19,20^ For instance, Devine et al.^21^ showed that in a sample of 100 subjects who had participated in at least two studies in the previous year, three-quarters reported concealing some health information from researchers in their lifetime to avoid exclusion from enrollment in a study. They had designed ways to hide their participation in concomitant studies, conceal underlying medical conditions, intake of concomitant medications, or substance abuse. Participation in multiple clinical trials, simultaneously or without respecting a sufficient “wash-out” interval between studies, exposes HVs to potential safety risks related to drug-drug interactions.^22^ It may also compromise the validity of study results,^23^ thereby exposing future patients to unforeseen safety and efficacy issues.

## Material and Methods

We aimed to document the number of HVs participating in studies, anywhere in the world, that involve the administration of medicines or biologicals (pharmaceutical products derived from biological sources), with no prospect of direct medical benefit.

For this, we accessed four data sources with the intent of progressively narrowing our scope and getting more detailed information, including three web-based databases. First, TrialTrove,^24^ a privately owned database that merges information on clinical trials from multiple sources around the world. Second, the clinicaltrials.gov ^25^ database from the U.S. National Library of Medicine, which includes studies from multiple countries in addition to the United States (this database is one of the sources of TrialTrove). Third, a country’s national registry, the Clinical Trials Registry of India.^26^ And, finally, internal data from Sanofi, a global research-based pharmaceutical company.

We accessed the three web-based databases on a single occasion during the same week of September 2017, in order to get a snapshot picture of the situation. For Sanofi, we collected data on studies performed globally during the years 2014, 2015, and 2016.

We used a variety of the available data filters of the web-based databases to focus only on planned or ongoing studies. For the clinicaltrials.gov database, we selected the following filters: Phase I, early Phase I, Recruiting, Not yet recruiting, Active, Not recruiting, Enrolling by invitation, Suspended, and Unknown status. To focus on studies involving HVs, we selected the term “healthy” as a filter in “condition/disease,” in order to capture mentions of both “healthy volunteer(s)” and “healthy subject(s).” Aiming for the same objectives for the TrialTrove database, we selected the following filters: Phase I, Open, Temporarily closed, Planned, and the term Healthy in Inclusion Criteria. For the Clinical Trials Registry of India, we selected the following filters: Phase I, Not yet recruiting, Suspended, Open to recruitment, and Healthy volunteers.

However, trying to obtain precise and homogeneous data from web-based databases turned out to be very difficult, for a variety of reasons.

The first difficulty stems from the ambiguity of terms such as “Phase I studies” and “healthy volunteers,” resulting in wide variations in the way these studies are reported in databases or referred to in the literature. Most people equate “studies in HVs” with “Phase I studies,” that is first administrations of new chemical entities to humans. However, as will be shown later in this paper, the large majority of studies in HVs do not concern new chemical entities, but rather registered drugs which are tested for bioequivalence or bioavailability, most often versus a generic version, as part of the registration process of the generic drug. “Phase I” is also largely used for studies performed in patients. In the field of oncology in particular, early studies of new drugs potentially too toxic to be administered to HVs involve cancer patients for whom direct benefit is not usually expected but cannot be entirely ruled out. An added complication is that the term “healthy volunteer” can be used to designate people taking part in trials of preventative vaccines. These people are undoubtedly healthy, at least regarding the disease under consideration, but they *can* expect a benefit from their participation, that of being, potentially, protected against a disease. Lastly, HVs also participate in studies with no administration of medicines such as social science studies, sample collections, medical imagery studies, etc.; these studies are outside the scope of this paper since participants are not, usually, exposed to a medical risk.

The second set of difficulties comes from the fact that data on clinical trials performed with HVs are largely owned by private corporations, i.e., pharmaceutical companies (research-based and generic) that sponsor most of these studies, and by CROs that carry them out. There is no central repository where all trials in HVs can be found. U.S. regulations for “applicable clinical trials” that must be registered in the “clinicaltrials.gov” database specifically exclude Phase 1 trials of a drug and/or biological product. There is nevertheless a section of the clinicaltrials.gov database dedicated to Phase I trials, where a large variety of trials are recorded. This seems to indicate that some sponsors decide to voluntarily post information on “first-in-human” studies (the usual definition of “Phase I” studies), while others use the term “Phase I” to report, for instance, any study where no direct therapeutic benefit to participants is expected, such as early studies of medicines or biologicals in cancer patients, as well as studies that are observational or focus on biological samples collections or medical imagery.

## Results: Available Data on Studies with Healthy Volunteers

The TrialTrove database yielded a total of 28,716 Phase I studies, performed at 13,547 sites around the world. Focusing on planned or ongoing Phase 1 trials yielded a total of 1,435 trials. **Table 1** shows the countries where these studies are reported to take place. North America, Western and Central Europe, and East Asia report the largest numbers of trials, but it is interesting to see that Phase 1 studies are carried out on all continents and in a large variety of countries.

**Table 1.** Planned and Ongoing Phase 1 Studies Reported in Two Web-Based Databases

	TrialTrove Database^42^	Clinicaltrials.gov Database^43^
World	1435	1085
Africa	13	27
	(3 South Africa, 2 Kenya, 2 Uganda, 1 Benin, 1 Burkina Faso, 1 Equatorial Guinea, 1 Egypt, 1 Gabon, 1 Mali, 1 Mozambique)	(10 South Africa, 4 Mali, 4 Kenya, 4 Zambia, 2 Burkina Faso, 2 Tanzania, 2 Uganda, 1 Benin, 1 Egypt, 1 Gabon, 1 Nigeria, 1 Rwanda, 1 Zimbabwe)
America		
North & Central America	677	421
	(367 Mexico, 289 USA, 21 Canada)	(388 USA, 31 Canada, 2 Mexico, 1 Puerto Rico, 1 Panama)
South America	9	27
	(7 Brazil, 2 Dominican Republic)	(21 Brazil, 2 Chile, 2 Uruguay, 1 Peru)
Asia		
East Asia	388	150
	(316 China, 62 Republic of Korea, 8 Taiwan)	(74 Republic of Korea, 51 China, 13 Taiwan, 3 Hong Kong)
South Asia	19	11
	(18 India, 1 Sri Lanka)	(7 India, 3 Bangladesh, 1 Pakistan)
Southeast Asia	31	17
	(18 Thailand, 7 Singapore, 5 Malaysia, 1 Indonesia)	(9 Singapore, 7 Thailand, 1 Malaysia, 1 Philippines)
Europe		
Eastern Europe	28	9
	(26 Russian Federation, 2 Moldova)	(5 Russian Federation, 4 Moldova, 2 Georgia)
Western and Central Europe	216	284
	(70 UK, 56 Germany, 23 Belgium, 14 Netherlands, 7 Denmark, 6 Bulgaria, 6 France, 5 Switzerland, 4 Austria, 4 Czech Republic, 4 Romania, 4 Sweden, 3 Spain, 2 Italy, 1 Finland, 1 Georgia, 1 Ireland, 1 Norway, 1 Poland, 1 Portugal)	(108 UK, 64 Germany, 39 Belgium, 20 Netherlands, 13 Switzerland, 11 Spain, 10 France, 9 Sweden, 10 Denmark, 6 Romania, 6 Bulgaria, 5 Austria, 3 Hungary, 3 Finland, 2 Poland, 2 Italy, 2 Sweden, 1 Portugal, 1 Slovakia)
Japan	22	15
Middle East	11	17
	(8 Israel, 2 Turkey, 1 Jordan)	(11 Israel, 5 Jordan, 1 Turkey)
Pacific	49	40
	(28 Australia, 21 New Zealand)	(30 Australia, 13 New Zealand)

Note: Numbers do not always add up; they are reported as they appear in the databases. Some adjustments in region allocation have been made to enable side-by-side comparisons between the two databases.

The U.S. clinicaltrials.gov database included a total of 250,494 studies, with no filter applied regarding study type or status (ongoing, completed, etc.). Out of these, 41,220 (16.5%) were reported as “Phase I studies,” and 11,832 were reported as planned or ongoing. Adding the term “healthy” as a “condition/disease” filter, 1,085 studies were identified. Overall, as shown in Table 1, the same distribution between continents and the same large variety of countries were found for both the TrialTrove and clinicaltrials.gov databases, with the notable exception of Mexico, for which the TrialTrove lists 367 trials while clinicaltrials.gov lists 2 trials.

We repeated the same exercise with the Clinical Trials Registry of India.^27^ Out of a total of 9,114 studies registered, 312 (3.4%) were labeled as “Phase I” and 26 (0.3%) were reported when “Phase I” and “healthy volunteers” filters were combined. In both instances, many studies appeared to involve patients. As was observed with the U.S. database, when the term “healthy volunteer” was used as a key word, a sizeable number of studies (225) were found, with the same wide variations in the types of studies reported. The low number of “Phase I” studies is consistent with the requirement of Indian authorities that first administrations to “humans” of new chemical entities be carried out in the country of origin of the new compound. Therefore, the overwhelming majority of studies can be expected to be pharmacokinetic studies, reflecting the large number of generic companies present in India.^28^

Given the difficulties of interpreting data extracted from databases with large amounts of data entered by a wide variety of persons, we saw the database of one large multinational research-based pharmaceutical company as a way to help shed light on the situation. At the request of the Sanofi Bioethics Committee, we obtained data on studies sponsored by Sanofi, involving HVs and administration of medicines, performed during the years 2014, 2015 and 2016. As shown in **Table 2**, a total of 122 studies were performed over this 3-year period, involving approximately 4,800 HVs. Out of these 122 studies, 113 (93%) were pharmacokinetic studies (bioavailability, bioequivalence, drug-drug interaction), and only 9 (7%) were “first-in-human” studies (single or multiple ascending doses of new chemical entities). These studies were performed in 15 different countries, with Canada, Brazil, and Romania heading the list. The choice of countries differed depending on the type of studies. For “first-in-human” studies, study sites were largely chosen based on the previous experience of Sanofi scientists regarding the level of expertise of the investigators, the ease of the procurement process (meaning that formal agreements were more readily made with institutions with which Sanofi had already worked), and regulatory and logistical issues such as requirements for study drug labeling and shipment. For pharmacokinetic studies, countries were most often chosen based on regulatory requirements to have local data provided for registration (e.g., Indonesia, Malaysia, Russia, South Africa). Many pharmacokinetic studies were performed in Canada for reasons related to the quality, cost, and speed of data provided by CROs based in that country, and in Brazil since Sanofi has a large generic branch in that country.

**Table 2.** Sanofi-Sponsored Studies with Administration of Medicines Involving Healthy Volunteers, Performed During the Years 2014, 2015, and 2016

Total Number of Studies Performed	Type and Number of Studies	Total Number of Healthy Volunteers Involved	Number of Studies Per Country	
122	Pharmacokinetic studies (bioavailability, bioequivalence, drug-drug interaction): 113 “First-in-human” studies of new chemical entities (single or multiple ascending doses): 9	Approximately 4800*	Canada Brazil Romania Czech Republic Germany USA/Canada France India Malaysia USA China Indonesia Turkey Bulgaria Japan Rep. of Korea South Africa	40 19 15 8 7 6 5 4 4 4 3 2 2 1 1 1 1

* Based on an average estimated number of 40 healthy volunteers per pharmacokinetic study.

## Discussion: The Need for Better Informed Consent and National Registries

Even though assessing the actual number of studies involving HVs globally is a challenge, the data we have found from a variety of sources consistently supports some clear conclusions: tens of thousands of HVs are involved every year in clinical trials in a large variety of countries on all five continents, and the overwhelming majority of these studies appear to be pharmacokinetic rather than “first-in-human” studies. We believe that HVs would be better protected by improving the way their informed consent process is managed and by setting up national registries.

To ensure that HVs properly understand the information that is provided to them during the informed consent process and are able to determine the level of risk to which they expose themselves, it can be proposed that tests be performed to check that each HV has properly understood the most critical information provided. The results of these tests should be documented, and HVs who do not demonstrate a sufficient understanding, possibly after a second round of information, should be excluded from participation. Such an approach will exclude HVs that are not able to understand basic scientific concepts and would therefore help ensuring proper informed consent.

National compulsory registries, where each HV must be registered before being considered for participation, can prevent individual HVs from participating in several trials at the same time and thereby ensure that they respect the required “wash-out” periods between trials. Such registries can also be used to track payments made to each individual so as to ensure that they do not go above an acceptable level that could jeopardize their ability to refuse participation. To our knowledge, France and the U.K. are the only countries where national registries have been set up.

France’s national registry, the *Volontaires Recherche Biomédicale*,^29^ is based on a law (*loi Huriet-Serusclat*), passed in late 1988, which established a legal framework for trials involving HVs.^30^ It is administered by the Ministry of Health, and its operating mode was most recently revised by law in 2006.^31^ Registration of HVs by investigators is mandatory, and HVs must be covered by the national “*Sécurité Sociale*” scheme. The information entered into the database includes the identity, date and place of birth of the HV, the dates of study participation, and, if appropriate, the poststudy exclusion period during which no other study participation is allowed. The amount of financial compensation received for each study is also entered, and a maximum level of earnings is set by law, currently 4,500 Euros over a 12-month period.

The U.K. national registry, the Over-Volunteering Prevention System “TOPS”,^32^ was initially operated on a voluntary basis and administered by an independent charity. It was initiated following the findings by the Hammersmith Medical Research Phase I unit in London that from 1997 to 2001, 1 in 10 healthy volunteers they screened had completed a study of a potential new medicine within the previous 12 weeks, even though they had signed a statement to the contrary.^33^ The same team has documented how the use of TOPS by an increasing number of U.K. Phase I units has resulted in decreasing the incidence of subjects attempting to volunteer within 3 months of completing another trial in another unit. Since 2013, TOPS has come within the remit of the National Health Service’s Health Research Authority, and registration of individual HVs has become a standard condition of ethical approval, as well as part of the Medicines & Healthcare Products Regulatory Agency accreditation scheme. HVs are identified by their National Insurance number (for U.K. citizens) or by their passport number and country of origin (for non-U.K. citizens). TOPS does not include information on post-study exclusion periods, only the date of the last dose of study medicine received. It does not include information on payments made to HVs.

The Swiss canton of Ticino also set up a mandatory registry for HVs in the early 2000s.^34^ Voluntary, privately managed registries have been set up in the USA and Canada: the Clinical Research Subject Verification Program^35^ and Verified Clinical Trials.^36^ U.S. researchers have advocated that a national registry could play an important role in promoting research integrity and protecting subjects from harm.^37,38,39,40^ National mandatory registries require clear operating rules and multiple safeguards to ensure, in particular, HV data privacy. They can only be set up by law, and the examples of France and the U.K. could help other countries to design similar systems.

## Conclusion

We have moved a long way since the early days of clinical pharmacology in the 1980s, when Phase I studies were carried out in a limited number of academic institutions in Europe and North America, and most HVs were university students, often from medical schools.

A large global industry has now been set up to obtain the data required by drug developers and regulatory agencies from studies in HVs. In this paper, it was shown how difficult it is to get a proper idea of the number of clinical trials involving HVs that are carried out around the world. But however limited the data we have collected, it is clear that every year thousands of clinical trials, involving tens of thousands of HVs, are carried out, partly for “first-in-human” studies, but overwhelmingly for pharmacokinetic studies related to the registration of generic medicines. It is also clear that everywhere in the world, most HVs are vulnerable people because of their economic circumstances and/or their literacy levels.

Setting up national HV registries can be an appropriate way to ensure that HVs are prevented from concealing their participation in multiple trials and receive appropriate financial indemnification that does not represent undue financial inducement. National HV registries should help to reduce the number of people participating in *multiple* clinical trials. Preventing poor people from earning a living out of participation in clinical trials may be seen as raising additional ethical concerns. As stated by Fisher,^41^ for some “the systematic use of economically disenfranchised people like healthy volunteers in pharmaceutical clinical development is not seen as being exploitative, but is instead presented as an opportunity for the members of these groups.” Nevertheless, the two basic tenets of ethical participation in clinical trials are proper informed consent and the avoidance of undue financial inducements that would negate the ability to freely accept or refuse participation. On both matters, no compromise should be accepted.

Most clinical trials involving HVs are sponsored by pharmaceutical firms, both generic and research-based, and performed by CROs. The standards used by major pharmaceutical companies for the clinical trials they sponsor contribute to raising the standards of ethical and scientific quality globally. Pharmaceutical companies and CROs should take pride in supporting initiatives to ensure that this neglected, highly vulnerable group of persons benefits from the best possible safeguards. National registries, as in France and in the U.K., can only be set up by governments’ decisions. It is the responsibility of all stakeholders involved in early clinical research, both private and public, to bring these issues and proposals to the attention of political decision makers.

## Notes

2. World Medical Association. WMA Declaration of Helsinki: Ethical principles for medical research involving human subjects; 2013; available at https://www.wma.net/policies-post/wma-declaration-of-helsinki-ethical-principles-for-medical-research-involving-human-subjects/ (last accessed 10 Oct 2017).10.1001/jama.2013.28105324141714

3. Council for International Organizations of Medical Sciences. International Ethical Guidelines for Health-Related Research Involving Humans; 2016; available at https://cioms.ch/wp-content/uploads/2017/01/WEB-CIOMS-EthicalGuidelines.pdf (last accessed 10 Oct 2017).40523065

4. StunkelL, GradyC. More than the money: A review of the literature examining healthy volunteer motivations. Contemporary Clinical Trials 2011;32:342–52.2114663510.1016/j.cct.2010.12.003PMC4943215

5. BigorraJ, BanosJE. Weight of financial reward in the decision by medical students and experienced healthy volunteers to participate in clinical trials. European Journal of Clinical Pharmacology. 1990;38(5):443–6.237952810.1007/BF02336681

6. DoshiMS, KulkarniSP, GhiaCJ, GogtayNJ, ThatteUM. Evaluation of factors that motivate participants to consent for non-therapeutic trials in India. Journal of Medical Ethics 2013;39(6):391–6. doi: 10.1136/medethics-2012-10075523475804

7. NappoSA, IafrateGB, SanchezSM. Motives for participating in a clinical research trial: A pilot study in Brazil. BMC Public Health 2013;13:19. doi: 10.1186/1471-2458-13-1923302375PMC3554489

8. AntonesiIM, RotaruOC, GhiciucCM. Ethical challenges of enrollment of healthy volunteers in pharmacometric studies. Romanian Journal of Bioethics 2012;10(2):122–30.

9. AlmeidaL, AzevedoB, NunesT, Vaz-da-SilvaM, Soares-da-SilvaP. Why healthy subjects volunteer for phase I studies and how they perceive their participation. European Journal of Clinical Pharmacology 2007;63:1085–94.1789153610.1007/s00228-007-0368-3

10. TishlerCL, BartholomaeS. The recruitment of normal healthy volunteers: A review of the literature on the use of financial incentives. Journal of Clinical Pharmacology 2002;42:365–75.11936560

11. See note 5, Bigorra, Banos 1990:443–6.

12. FisherJA. Feeding and bleeding: The institutional banalization of risk to healthy volunteers in phase I pharmaceutical clinical trials. Science Technology & Human Values 2015 March 1;40(2):199–226.10.1177/0162243914554838PMC440579325914430

13. FisherJA. Coming soon to a physician near you: Medical neoliberalism and pharmaceutical clinical trials. Harvard Health Policy Review 2007;8(1):61–70.21572938PMC3092550

14. ElliottC, AbadieR. Exploiting a research underclass in phase 1 clinical trials. New England Medical Journal 2008;358(22):2316–17.10.1056/NEJMp080187218509119

15. RabinC, TabakN. Healthy participants in phase I clinical trials: The quality of their decision to take part. Journal of Clinical Nursing 2006;15:971–9.1687954110.1111/j.1365-2702.2006.01388.x

16. KrishnaS, Purendra PrasadN. Ethical issues in recruitment of “healthy volunteers”: Study of a clinical research organisation in Hyderabad. Indian Journal of Medical Ethics 2014;4:228–32.10.20529/IJME.2014.05725377035

17. See note 16, Krishna, Purendra Prasad 2014.

18. MotlukA. Perils of the professional lab rat. New Scientist 2009;2718:40–3.

19. EdelbluteHB, FisherJA. Using “clinical trial diaries” to track patterns of participation for serial healthy volunteers in U.S. phase I studies. Journal of Empirical Research on Human Research Ethics 2015;10(1):65–75. doi:10.1177/155626461456828025742668PMC4408988

20. MonahanT, FisherJA. ‘I’m still a hustler’: Entrepreneurial responses to precarity by participants in phase I clinical trials. Economics and Society 2015;44(4):545–66. doi: 10.1080/03085147.2015.1113703PMC497813127524854

21. DevineEG, WatersME, PutnamM, SurpriseC, O’MalleyK, RichambaultC, et al Concealment and fabrication by experienced research subjects. Clinical Trials 2013;10:935–48.2386722310.1177/1740774513492917

22. ResnikDB, McCannDJ. Deception by research participants. New England Journal of Medicine 2015;373(13):1192–3.2639806910.1056/NEJMp1506985

23. ResnikDB, KoskiG. A national registry for healthy volunteers in phase 1 clinical trials. JAMA 2011;305:1236–7.2140663610.1001/jama.2011.354PMC3094908

24. Pharma Intelligence. Sitetrove: Clinical Trial Site and Investigator Identification; 2017; available at https://pharmaintelligence.informa.com/products-and-services/data-and-analysis/sitetrove (last accessed 9 June 2017).

25. ClinicalTrials.gov. A Service of the US National Institutes of Health; available at https://clinicaltrials.gov/ct2/home (last accessed 6 Sept 2017).

26. Clinical Trials Registry-India. *National Institute of Medical Statistics (Indian Council of Medical Research)*; available at http://ctri.nic.in/Clinicaltrials/login.php (last accessed 6 Sep 2017).

27. See note 26, Clinical Trials Registry-India.

28. Mondal S, Abrol D. *Clinical Trial Industry in India: A Systematic Review*. New Delhi: Institute for Studies in Industrial Development working paper 179; 2015.

29. *Registre Volontaires Recherches Biomédicales*. available at https://vrb.sante.gouv.fr/vrb/ (last accessed 10 Nov 2017).

30. Loi n° 88-1138 du 20 décembre 1988 relative à la protection des personnes qui se prêtent à des recherches biomédicales. *Journal Officiel de la République Française*; 1988; available at https://www.legifrance.gouv.fr/affichTexte.do?cidTexte=JORFTEXT000000508831 (last accessed 10 Nov 2017).

31. Arrêté du 14 novembre 2006 relatif aux données constitutives du fichier national des personnes qui se prêtent à des recherches biomédicales. *Journal Officiel de la République Française* n°291 2006 Dec:19015, texte n° 39. available at https://www.legifrance.gouv.fr/affichTexte.do?cidTexte=JORFTEXT000000646248&dateTexte=&categorieLien=id#B# (last accessed 10 Oct 2017).

32. Health Research Authority. *The Over-Volunteering Prevention System (TOPS)*; available at http://www.hra.nhs.uk/about-the-hra/our-committees/the-over-volunteering-prevention-system/ (last accessed 12 Oct 2017).

33. BoyceM, NentwichH, MelbourneW, WarringtonS. TOPS: the over-volunteering prevention system. British Journal of Clinical Pharmacology. 2003;55:643. doi: 10.1046/j.1365-2125.2003.01923

34. ZaniniGM, MaroneC. A new job: Research volunteer? Swiss Medical Weekly 2005;135:315–7.1603468510.4414/smw.2005.10956

35. Clinicalrsvp.com. *Clinical Research Subject Verification Program*. c2012 [cited 2017 Sept 12]; available at http://www.clinicalrsvp.com/clinicalRSVPOverview.aspx.

36. Verifiedclinicaltrials.com [Internet]. c2017 [cited 2017 Oct 7]; available at http://www.verifiedclinicaltrials.com/.

37. Kupetsky-RinconEA, KraftWK. Healthy volunteer registries and ethical research principles. Clinical Pharmacology & Therapeutics 2012;91(6):965–8. doi:10.1038/clpt.2012.3222609908PMC5164923

38. See note 23, Resnik , Koski 2011.

39. See note 22, Resnik, McCann 2015.

40. See note 19, Edelblute, Fisher 2015.

41. See note 13, Fisher 2007.

42. See note 24, Pharma Intelligence.

43. See note 25, ClinicalTrials.gov.

